# 1,3-Bis(biphenyl-4-yl)-2,2-dibromo-3-oxopropyl acetate

**DOI:** 10.1107/S1600536811056169

**Published:** 2012-01-11

**Authors:** Jerry P. Jasinski, James A. Golen, B. P. Siddaraju, B. Narayana, H. S. Yathirajan

**Affiliations:** a Department of Chemistry, Keene State College, 229 Main Street, Keene, NH 03435-2001, USA; b Department of Studies in Chemistry, University of Mysore, Manasagangotri, Mysore 570 006, India; c Department of Studies in Chemistry, Mangalore University, Mangalagangotri, 574 199, India

## Abstract

In the title compound, C_29_H_22_Br_2_O_3_, the dihedral angles between the mean planes of the benzene rings within each biphenyl group are 26.7 (8) and 30.9 (8)°. The mean planes of the terminal and inner benzene rings of the biphenyl groups bonded through a propan-1-one group in the V-shaped mol­ecule are oriented at angles of 66.1 (7) and 60.0 (8)°, respectively. The two Br atoms are opposite the propen-1-one group. Weak inter­molecular C—H⋯O and C—H⋯π inter­actions are observed in the crystal structure.

## Related literature

For chalcone derivatives exhibiting non-linear optical effects, see: Indira *et al.* (2002 ▶); Tam *et al.* (1989 ▶); Uchida *et al.* (1998 ▶). For the improvement of mol­ecular first-order hyperpolarizabilities, see: Zhao *et al.* (2002 ▶). For related dibromo chalcone structures, see: Butcher *et al.* (2007 ▶); Narayana *et al.* (2007 ▶); Sarojini *et al.* (2007 ▶); Yathirajan *et al.* (2007 ▶); For the synthesis of various chalcone derivatives, see: Samshuddin *et al.* (2011 ▶); Jasinski *et al.* (2010 ▶).
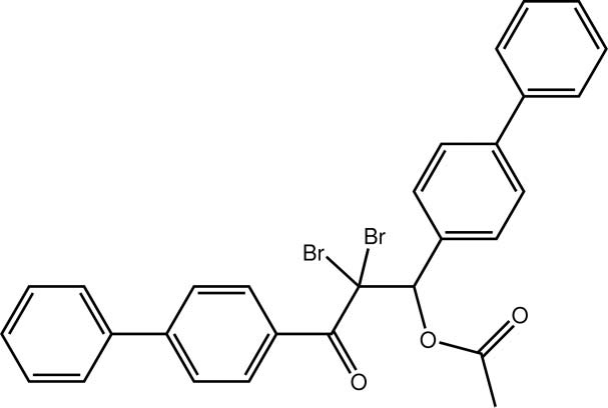


## Experimental

### 

#### Crystal data

C_29_H_22_Br_2_O_3_
*M* *_r_* = 578.29
Monoclinic, 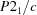

*a* = 12.0497 (14) Å
*b* = 20.842 (2) Å
*c* = 9.9482 (10) Å
β = 98.743 (10)°
*V* = 2469.4 (5) Å^3^
*Z* = 4
Mo *K*α radiation
μ = 3.31 mm^−1^
*T* = 173 K
0.20 × 0.20 × 0.10 mm

#### Data collection

Oxford Diffraction Xcalibur Eos Gemini diffractometer
Absorption correction: multi-scan (*CrysAlis RED*; Oxford Diffraction, 2010 ▶) *T*_min_ = 0.557, *T*_max_ = 0.733
22652 measured reflections
5881 independent reflections
3640 reflections with *I* > 2σ(*I*)
*R*_int_ = 0.068

#### Refinement

*R*[*F* ^2^ > 2σ(*F* ^2^)] = 0.052
*wR*(*F* ^2^) = 0.130
*S* = 1.02
5881 reflections
308 parameters
H-atom parameters constrained
Δρ_max_ = 0.72 e Å^−3^
Δρ_min_ = −0.59 e Å^−3^

### 

Data collection: *CrysAlis PRO* (Oxford Diffraction, 2010 ▶); cell refinement: *CrysAlis PRO*; data reduction: *CrysAlis RED* (Oxford Diffraction, 2010 ▶); program(s) used to solve structure: *SHELXS97* (Sheldrick, 2008 ▶); program(s) used to refine structure: *SHELXL97* (Sheldrick, 2008 ▶); molecular graphics: *SHELXTL* (Sheldrick, 2008 ▶); software used to prepare material for publication: *SHELXTL*.

## Supplementary Material

Crystal structure: contains datablock(s) global, I. DOI: 10.1107/S1600536811056169/tk5043sup1.cif

Structure factors: contains datablock(s) I. DOI: 10.1107/S1600536811056169/tk5043Isup2.hkl

Supplementary material file. DOI: 10.1107/S1600536811056169/tk5043Isup3.cml

Additional supplementary materials:  crystallographic information; 3D view; checkCIF report

## Figures and Tables

**Table 1:** Hydrogen-bond geometry (Å, °) *Cg*4 is the centroid of the C24–C29 ring.

*D*—H⋯*A*	*D*—H	H⋯*A*	*D*⋯*A*	*D*—H⋯*A*
C1—H1*C*⋯O1^i^	0.98	2.41	3.336 (6)	158
C17—H17*A*⋯O3^ii^	0.95	2.47	3.369 (5)	158
C20—H20*A*⋯*Cg*4^iii^	0.95	2.82	3.707 (4)	157

Symmetry codes: (i) 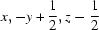; (ii) 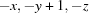; (iii) 
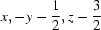
.

**Fig. 1.**
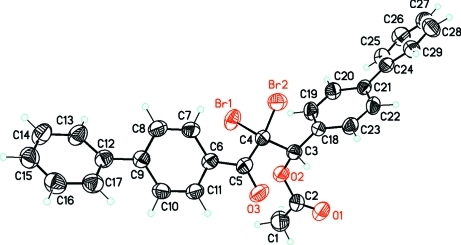
Molecular structure of the title compound showing the atom labeling scheme and 50% probability displacement ellipsoids.

**Fig. 2.**
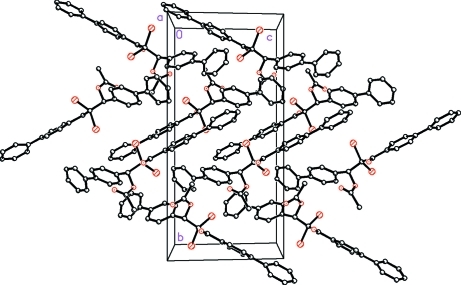
Packing diagram of the title compound viewed along the a axis. Hydrogen atoms have been omitted for clarity.

## supplementary crystallographic information

### Comment

Among several organic compounds exhibiting NLO effects, chalcone derivatives are important materials for their excellent blue light transmittance and good crystallizability. It has been observed that substitution of a bromo group on either of the phenyl rings greatly influences non-centrosymmetric crystal packing (Uchida *et al.*, 1998; Tam *et al.*, 1989; Indira *et al.*, 2002). Bromo substituents can obviously improve molecular first-order hyperpolarizabilities and can effectively reduce dipole-dipole interactions between molecules (Zhao *et al.*, 2002). Chalcone derivatives usually have lower melting points, which can be a drawback when their crystals are used in optical instruments. Chalcone dibromides usually have higher melting points and are thermally stable. In order to synthesize the dibromo derivative of this chalcone, (2*E*)-1,3-di(biphenyl-4-yl)prop-2-en-1-one was brominated using bromine in acetic acid. But instead of the dibromo derivative of this chalcone, a new product 2,2-dibromo-1,3-di(biphenyl-4-yl)-3-oxopropyl acetate (I) has been obtained.

The crystal structures of some dibromo chalcones viz., 2,3-dibromo-3-(5-bromo-2-methoxyphenyl)-1-(2,4-dichlorophenyl) propan-1-one (Narayana *et al.*, 2007), 2,3-dibromo-3-(4-bromo-6-methoxy -2-naphthyl)-1-(4-methoxyphenyl)propan-1-one (Sarojini *et al.*, 2007), 2,3-dibromo-3-(5-bromo-6-methoxy-2-naphthyl)-1-(2,4-dichlorophenyl) propan-1-one (Yathirajan *et al.*, 2007) and (2Z)-2-bromo-3-[3,5-dibromo-4-(ethylamino)phenyl]-1- (2,4-dichlorophenyl)prop-2-en-1-one (Butcher *et al.*, 2007) have been reported. In continuation of our work on synthesis of various derivatives of chalcone (Samshuddin *et al.*, 2011; Jasinski *et al.*, 2010), the title chalcone dibromide, (I), was prepared and its crystal structure is reported.

In the crystal structure of (I), the dihedral angles between the mean planes of the benzene rings within each biphenyl group are 26.7 (8)° and 30.9 (8)° (Fig. 1). The mean planes of the terminal and inner benzene rings of the biphenyl groups bonded through a propan-1-one group in the V-shaped molecule are oriented at angles of 66.1 (7) and 60.0 (8)°, respectively. The two bromine atoms are opposite the propen-1-one group extending in an apical configuration. Weak C—H···O and C—H···Cg π-ring intermolecular interactions are observed in the crystal structure (Table 1, Fig. 2).

### Experimental

To a solution of (2*E*)-1,3-di(biphenyl-4-yl)prop-2-en-1-one (3.60 g, 0.01 mol) in acetic acid (25 ml), bromine (1.60 g, 0.01 mol) in acetic acid (10 ml) was added slowly with stirring at 273 K. After completion of the addition of the bromine solution, the reaction mixture was stirred for 5 h. The solid obtained was filtered and recrystallized from acetone. Single crystals were grown from its methanol solution by slow evaporation. The yield of the compound was 86%. (m.p.: 445 K).

### Refinement

All of the H atoms were placed in their calculated positions and then refined using the riding model with C—H lengths of 0.95–1.00 Å (CH) or 0.98 Å (CH_3_). Isotropic displacement parameters for these atoms were set to 1.19–1.20 (CH) or 1.49 (CH_3_) × *U*_eq_ of the parent atom.

### Crystal data

**Table:**

C_29_H_22_Br_2_O_3_	*F*(000) = 1160
*M**_r_* = 578.29	*D*_x_ = 1.555 Mg m^−^^3^
Monoclinic, *P*2_1_/*c*	Mo *K*α radiation, λ = 0.71073 Å
Hall symbol: -P 2ybc	Cell parameters from 2960 reflections
*a* = 12.0497 (14) Å	θ = 3.0–30.0°
*b* = 20.842 (2) Å	µ = 3.31 mm^−^^1^
*c* = 9.9482 (10) Å	*T* = 173 K
β = 98.743 (10)°	Block, colourless
*V* = 2469.4 (5) Å^3^	0.20 × 0.20 × 0.10 mm
*Z* = 4	

### Data collection

**Table:**

Oxford Diffraction Xcalibur Eos Gemini diffractometer	5881 independent reflections
Radiation source: Enhance (Mo) X-ray Source	3640 reflections with *I* > 2σ(*I*)
graphite	*R*_int_ = 0.068
Detector resolution: 16.1500 pixels mm^-1^	θ_max_ = 27.9°, θ_min_ = 3.0°
ω scans	*h* = −15→15
Absorption correction: multi-scan (*CrysAlis RED*; Oxford Diffraction, 2010)	*k* = −27→26
*T*_min_ = 0.557, *T*_max_ = 0.733	*l* = −13→12
22652 measured reflections	

### Refinement

**Table:**

Refinement on *F*^2^	Primary atom site location: structure-invariant direct methods
Least-squares matrix: full	Secondary atom site location: difference Fourier map
*R*[*F*^2^ > 2σ(*F*^2^)] = 0.052	Hydrogen site location: inferred from neighbouring sites
*wR*(*F*^2^) = 0.130	H-atom parameters constrained
*S* = 1.02	*w* = 1/[σ^2^(*F*_o_^2^) + (0.053*P*)^2^ + 0.4853*P*] where *P* = (*F*_o_^2^ + 2*F*_c_^2^)/3
5881 reflections	(Δ/σ)_max_ = 0.001
308 parameters	Δρ_max_ = 0.72 e Å^−^^3^
0 restraints	Δρ_min_ = −0.59 e Å^−^^3^

### Special details

**Table:**

Geometry. All e.s.d.'s (except the e.s.d. in the dihedral angle between two l.s. planes) are estimated using the full covariance matrix. The cell e.s.d.'s are taken into account individually in the estimation of e.s.d.'s in distances, angles and torsion angles; correlations between e.s.d.'s in cell parameters are only used when they are defined by crystal symmetry. An approximate (isotropic) treatment of cell e.s.d.'s is used for estimating e.s.d.'s involving l.s. planes.
Refinement. Refinement of *F*^2^ against ALL reflections. The weighted *R*-factor *wR* and goodness of fit *S* are based on *F*^2^, conventional *R*-factors *R* are based on *F*, with *F* set to zero for negative *F*^2^. The threshold expression of *F*^2^ > σ(*F*^2^) is used only for calculating *R*-factors(gt) *etc*. and is not relevant to the choice of reflections for refinement. *R*-factors based on *F*^2^ are statistically about twice as large as those based on *F*, and *R*- factors based on ALL data will be even larger.

### Fractional atomic coordinates and isotropic or equivalent isotropic displacement parameters (Å^2^)

**Table:**

	*x*	*y*	*z*	*U*_iso_*/*U*_eq_	
Br1	0.44982 (4)	0.34440 (2)	0.13153 (5)	0.05921 (17)	
Br2	0.42924 (4)	0.46937 (2)	0.31413 (5)	0.05352 (15)	
O1	0.1796 (3)	0.28133 (16)	0.4651 (3)	0.0722 (10)	
O2	0.3020 (2)	0.28697 (12)	0.3156 (3)	0.0488 (7)	
O3	0.1617 (2)	0.39172 (18)	0.2153 (3)	0.0758 (10)	
C1	0.1816 (5)	0.1984 (2)	0.2981 (5)	0.0736 (15)	
H1A	0.1179	0.1800	0.3354	0.110*	
H1B	0.2442	0.1679	0.3100	0.110*	
H1C	0.1592	0.2073	0.2010	0.110*	
C2	0.2174 (4)	0.2594 (2)	0.3710 (5)	0.0539 (11)	
C3	0.3430 (3)	0.34764 (17)	0.3725 (4)	0.0406 (9)	
H3A	0.2813	0.3675	0.4151	0.049*	
C4	0.3582 (3)	0.38888 (19)	0.2475 (4)	0.0423 (9)	
C5	0.2436 (3)	0.4071 (2)	0.1659 (4)	0.0484 (10)	
C6	0.2285 (3)	0.44157 (18)	0.0336 (4)	0.0418 (9)	
C7	0.3135 (3)	0.46850 (19)	−0.0303 (4)	0.0480 (10)	
H7A	0.3894	0.4656	0.0125	0.058*	
C8	0.2891 (3)	0.4989 (2)	−0.1532 (4)	0.0496 (10)	
H8A	0.3484	0.5168	−0.1939	0.060*	
C9	0.1792 (3)	0.50414 (19)	−0.2200 (4)	0.0432 (9)	
C10	0.0950 (3)	0.47780 (18)	−0.1548 (4)	0.0454 (10)	
H10A	0.0189	0.4809	−0.1969	0.054*	
C11	0.1194 (3)	0.44754 (19)	−0.0316 (4)	0.0452 (10)	
H11A	0.0599	0.4303	0.0099	0.054*	
C12	0.1512 (3)	0.53706 (17)	−0.3519 (4)	0.0435 (9)	
C13	0.2239 (4)	0.5370 (2)	−0.4479 (5)	0.0557 (12)	
H13A	0.2932	0.5147	−0.4287	0.067*	
C14	0.1977 (4)	0.5685 (2)	−0.5701 (5)	0.0600 (12)	
H14A	0.2495	0.5680	−0.6332	0.072*	
C15	0.0983 (4)	0.6004 (2)	−0.6020 (5)	0.0567 (11)	
H15A	0.0810	0.6224	−0.6862	0.068*	
C16	0.0235 (4)	0.6000 (2)	−0.5100 (5)	0.0571 (12)	
H16A	−0.0467	0.6213	−0.5318	0.069*	
C17	0.0496 (4)	0.5689 (2)	−0.3860 (5)	0.0525 (11)	
H17A	−0.0027	0.5694	−0.3236	0.063*	
C18	0.4403 (3)	0.33835 (17)	0.4820 (4)	0.0380 (9)	
C19	0.5332 (3)	0.30086 (19)	0.4645 (4)	0.0485 (10)	
H19A	0.5355	0.2805	0.3795	0.058*	
C20	0.6214 (3)	0.29298 (19)	0.5683 (4)	0.0470 (10)	
H20A	0.6844	0.2683	0.5526	0.056*	
C21	0.6208 (3)	0.32024 (17)	0.6958 (4)	0.0392 (9)	
C22	0.5258 (3)	0.35519 (19)	0.7145 (4)	0.0463 (10)	
H22A	0.5208	0.3729	0.8014	0.056*	
C23	0.4388 (3)	0.36451 (19)	0.6088 (4)	0.0453 (10)	
H23A	0.3762	0.3897	0.6240	0.054*	
C24	0.7180 (3)	0.31323 (18)	0.8042 (4)	0.0417 (9)	
C25	0.7914 (4)	0.2617 (2)	0.8065 (5)	0.0554 (11)	
H25A	0.7759	0.2287	0.7405	0.066*	
C26	0.8855 (4)	0.2576 (2)	0.9022 (5)	0.0634 (13)	
H26A	0.9348	0.2222	0.9007	0.076*	
C27	0.9099 (4)	0.3037 (3)	0.9998 (5)	0.0687 (14)	
H27A	0.9757	0.3007	1.0656	0.082*	
C28	0.8380 (4)	0.3540 (2)	1.0011 (5)	0.0645 (13)	
H28A	0.8529	0.3857	1.0701	0.077*	
C29	0.7442 (4)	0.3597 (2)	0.9043 (5)	0.0527 (11)	
H29A	0.6965	0.3959	0.9057	0.063*	

### Atomic displacement parameters (Å^2^)

**Table:**

	*U*^11^	*U*^22^	*U*^33^	*U*^12^	*U*^13^	*U*^23^
Br1	0.0624 (3)	0.0713 (3)	0.0495 (3)	0.0171 (2)	0.0263 (2)	0.0032 (2)
Br2	0.0479 (3)	0.0544 (3)	0.0586 (3)	−0.0059 (2)	0.0094 (2)	−0.0019 (2)
O1	0.073 (2)	0.095 (2)	0.054 (2)	−0.0222 (19)	0.0280 (18)	−0.0089 (18)
O2	0.0503 (17)	0.0525 (16)	0.0455 (17)	−0.0068 (13)	0.0136 (14)	−0.0048 (13)
O3	0.0397 (17)	0.131 (3)	0.060 (2)	0.0013 (18)	0.0162 (16)	0.032 (2)
C1	0.091 (4)	0.069 (3)	0.060 (3)	−0.025 (3)	0.011 (3)	−0.001 (3)
C2	0.055 (3)	0.067 (3)	0.039 (3)	−0.008 (2)	0.007 (2)	0.007 (2)
C3	0.038 (2)	0.045 (2)	0.040 (2)	−0.0017 (18)	0.0107 (18)	−0.0078 (18)
C4	0.036 (2)	0.056 (2)	0.036 (2)	0.0000 (18)	0.0115 (17)	−0.0035 (18)
C5	0.039 (2)	0.065 (3)	0.043 (3)	−0.001 (2)	0.0129 (19)	−0.001 (2)
C6	0.038 (2)	0.047 (2)	0.042 (2)	−0.0016 (18)	0.0113 (18)	−0.0012 (18)
C7	0.032 (2)	0.061 (3)	0.051 (3)	0.0008 (19)	0.0057 (19)	0.006 (2)
C8	0.040 (2)	0.056 (3)	0.054 (3)	−0.0046 (19)	0.012 (2)	0.006 (2)
C9	0.043 (2)	0.046 (2)	0.042 (2)	−0.0016 (18)	0.0082 (18)	−0.0010 (18)
C10	0.035 (2)	0.054 (2)	0.047 (3)	−0.0046 (18)	0.0057 (19)	−0.0062 (19)
C11	0.038 (2)	0.051 (2)	0.048 (3)	−0.0071 (18)	0.0116 (19)	−0.0028 (19)
C12	0.044 (2)	0.041 (2)	0.047 (3)	0.0001 (18)	0.0087 (19)	0.0019 (18)
C13	0.051 (3)	0.062 (3)	0.057 (3)	0.012 (2)	0.018 (2)	0.015 (2)
C14	0.063 (3)	0.064 (3)	0.058 (3)	0.000 (2)	0.022 (2)	0.014 (2)
C15	0.062 (3)	0.052 (3)	0.053 (3)	−0.004 (2)	0.001 (2)	0.011 (2)
C16	0.051 (3)	0.053 (3)	0.064 (3)	0.008 (2)	0.000 (2)	0.006 (2)
C17	0.046 (3)	0.055 (3)	0.057 (3)	0.002 (2)	0.009 (2)	−0.003 (2)
C18	0.036 (2)	0.045 (2)	0.033 (2)	−0.0052 (17)	0.0063 (17)	−0.0016 (16)
C19	0.055 (3)	0.055 (2)	0.037 (2)	0.006 (2)	0.010 (2)	−0.0086 (19)
C20	0.043 (2)	0.052 (2)	0.047 (3)	0.0102 (19)	0.011 (2)	−0.0024 (19)
C21	0.044 (2)	0.038 (2)	0.037 (2)	−0.0034 (17)	0.0111 (18)	0.0016 (16)
C22	0.055 (3)	0.051 (2)	0.035 (2)	0.001 (2)	0.014 (2)	−0.0062 (18)
C23	0.043 (2)	0.051 (2)	0.044 (3)	0.0053 (18)	0.014 (2)	−0.0031 (19)
C24	0.045 (2)	0.041 (2)	0.039 (2)	−0.0053 (18)	0.0082 (18)	0.0066 (17)
C25	0.066 (3)	0.050 (2)	0.050 (3)	0.004 (2)	0.007 (2)	0.001 (2)
C26	0.056 (3)	0.070 (3)	0.063 (3)	0.012 (2)	0.004 (3)	0.017 (3)
C27	0.064 (3)	0.080 (4)	0.058 (3)	−0.006 (3)	−0.004 (3)	0.016 (3)
C28	0.071 (3)	0.064 (3)	0.054 (3)	−0.013 (3)	−0.005 (3)	−0.005 (2)
C29	0.056 (3)	0.052 (2)	0.049 (3)	−0.003 (2)	0.003 (2)	−0.002 (2)

### Geometric parameters (Å, °)

**Table:**

Br1—C4	1.949 (3)	C14—C15	1.364 (6)
Br2—C4	1.953 (4)	C14—H14A	0.9500
O1—C2	1.192 (5)	C15—C16	1.379 (6)
O2—C2	1.359 (5)	C15—H15A	0.9500
O2—C3	1.441 (4)	C16—C17	1.386 (6)
O3—C5	1.211 (4)	C16—H16A	0.9500
C1—C2	1.493 (6)	C17—H17A	0.9500
C1—H1A	0.9800	C18—C23	1.377 (5)
C1—H1B	0.9800	C18—C19	1.398 (5)
C1—H1C	0.9800	C19—C20	1.374 (6)
C3—C18	1.487 (5)	C19—H19A	0.9500
C3—C4	1.545 (5)	C20—C21	1.391 (5)
C3—H3A	1.0000	C20—H20A	0.9500
C4—C5	1.539 (6)	C21—C22	1.393 (5)
C5—C6	1.486 (6)	C21—C24	1.473 (6)
C6—C11	1.381 (5)	C22—C23	1.381 (6)
C6—C7	1.402 (5)	C22—H22A	0.9500
C7—C8	1.369 (6)	C23—H23A	0.9500
C7—H7A	0.9500	C24—C25	1.389 (6)
C8—C9	1.392 (6)	C24—C29	1.391 (6)
C8—H8A	0.9500	C25—C26	1.368 (6)
C9—C10	1.398 (5)	C25—H25A	0.9500
C9—C12	1.474 (6)	C26—C27	1.365 (7)
C10—C11	1.370 (6)	C26—H26A	0.9500
C10—H10A	0.9500	C27—C28	1.362 (7)
C11—H11A	0.9500	C27—H27A	0.9500
C12—C17	1.389 (6)	C28—C29	1.374 (6)
C12—C13	1.390 (5)	C28—H28A	0.9500
C13—C14	1.375 (6)	C29—H29A	0.9500
C13—H13A	0.9500		
			
C2—O2—C3	116.5 (3)	C15—C14—C13	120.9 (4)
C2—C1—H1A	109.5	C15—C14—H14A	119.5
C2—C1—H1B	109.5	C13—C14—H14A	119.5
H1A—C1—H1B	109.5	C14—C15—C16	118.8 (4)
C2—C1—H1C	109.5	C14—C15—H15A	120.6
H1A—C1—H1C	109.5	C16—C15—H15A	120.6
H1B—C1—H1C	109.5	C15—C16—C17	120.7 (4)
O1—C2—O2	123.7 (4)	C15—C16—H16A	119.7
O1—C2—C1	126.3 (4)	C17—C16—H16A	119.7
O2—C2—C1	110.0 (4)	C16—C17—C12	120.9 (4)
O2—C3—C18	111.0 (3)	C16—C17—H17A	119.6
O2—C3—C4	104.4 (3)	C12—C17—H17A	119.6
C18—C3—C4	119.0 (3)	C23—C18—C19	117.2 (4)
O2—C3—H3A	107.3	C23—C18—C3	120.1 (3)
C18—C3—H3A	107.3	C19—C18—C3	122.6 (3)
C4—C3—H3A	107.3	C20—C19—C18	121.0 (4)
C5—C4—C3	110.8 (3)	C20—C19—H19A	119.5
C5—C4—Br1	110.4 (3)	C18—C19—H19A	119.5
C3—C4—Br1	111.0 (3)	C19—C20—C21	121.7 (4)
C5—C4—Br2	106.2 (3)	C19—C20—H20A	119.1
C3—C4—Br2	107.7 (3)	C21—C20—H20A	119.1
Br1—C4—Br2	110.53 (17)	C20—C21—C22	117.0 (4)
O3—C5—C6	119.3 (4)	C20—C21—C24	120.9 (3)
O3—C5—C4	116.2 (4)	C22—C21—C24	122.1 (4)
C6—C5—C4	124.5 (3)	C23—C22—C21	121.1 (4)
C11—C6—C7	117.4 (4)	C23—C22—H22A	119.5
C11—C6—C5	116.0 (3)	C21—C22—H22A	119.5
C7—C6—C5	126.6 (4)	C18—C23—C22	121.9 (4)
C8—C7—C6	121.2 (4)	C18—C23—H23A	119.1
C8—C7—H7A	119.4	C22—C23—H23A	119.1
C6—C7—H7A	119.4	C25—C24—C29	116.9 (4)
C7—C8—C9	121.5 (4)	C25—C24—C21	121.4 (4)
C7—C8—H8A	119.3	C29—C24—C21	121.6 (4)
C9—C8—H8A	119.3	C26—C25—C24	121.2 (4)
C8—C9—C10	116.9 (4)	C26—C25—H25A	119.4
C8—C9—C12	122.3 (3)	C24—C25—H25A	119.4
C10—C9—C12	120.8 (4)	C27—C26—C25	121.2 (5)
C11—C10—C9	121.6 (4)	C27—C26—H26A	119.4
C11—C10—H10A	119.2	C25—C26—H26A	119.4
C9—C10—H10A	119.2	C28—C27—C26	118.7 (5)
C10—C11—C6	121.4 (4)	C28—C27—H27A	120.7
C10—C11—H11A	119.3	C26—C27—H27A	120.7
C6—C11—H11A	119.3	C27—C28—C29	121.1 (5)
C17—C12—C13	117.1 (4)	C27—C28—H28A	119.5
C17—C12—C9	120.9 (4)	C29—C28—H28A	119.5
C13—C12—C9	121.9 (4)	C28—C29—C24	121.0 (4)
C14—C13—C12	121.5 (4)	C28—C29—H29A	119.5
C14—C13—H13A	119.2	C24—C29—H29A	119.5
C12—C13—H13A	119.2		
			
C3—O2—C2—O1	−2.8 (6)	C17—C12—C13—C14	1.6 (7)
C3—O2—C2—C1	177.9 (4)	C9—C12—C13—C14	−179.2 (4)
C2—O2—C3—C18	93.1 (4)	C12—C13—C14—C15	−0.8 (7)
C2—O2—C3—C4	−137.5 (3)	C13—C14—C15—C16	−0.7 (7)
O2—C3—C4—C5	70.3 (4)	C14—C15—C16—C17	1.3 (7)
C18—C3—C4—C5	−165.3 (3)	C15—C16—C17—C12	−0.4 (7)
O2—C3—C4—Br1	−52.7 (3)	C13—C12—C17—C16	−1.0 (6)
C18—C3—C4—Br1	71.7 (4)	C9—C12—C17—C16	179.8 (4)
O2—C3—C4—Br2	−173.9 (2)	O2—C3—C18—C23	−126.1 (4)
C18—C3—C4—Br2	−49.5 (4)	C4—C3—C18—C23	112.8 (4)
C3—C4—C5—O3	5.7 (5)	O2—C3—C18—C19	50.6 (5)
Br1—C4—C5—O3	129.2 (4)	C4—C3—C18—C19	−70.5 (5)
Br2—C4—C5—O3	−111.0 (4)	C23—C18—C19—C20	−2.8 (6)
C3—C4—C5—C6	−174.3 (3)	C3—C18—C19—C20	−179.5 (4)
Br1—C4—C5—C6	−50.9 (5)	C18—C19—C20—C21	1.9 (6)
Br2—C4—C5—C6	69.0 (4)	C19—C20—C21—C22	1.0 (6)
O3—C5—C6—C11	−6.8 (6)	C19—C20—C21—C24	−177.7 (4)
C4—C5—C6—C11	173.2 (4)	C20—C21—C22—C23	−2.8 (6)
O3—C5—C6—C7	172.9 (4)	C24—C21—C22—C23	175.8 (4)
C4—C5—C6—C7	−7.1 (6)	C19—C18—C23—C22	0.9 (6)
C11—C6—C7—C8	−0.7 (6)	C3—C18—C23—C22	177.8 (4)
C5—C6—C7—C8	179.6 (4)	C21—C22—C23—C18	1.9 (6)
C6—C7—C8—C9	−0.3 (6)	C20—C21—C24—C25	−25.7 (5)
C7—C8—C9—C10	1.0 (6)	C22—C21—C24—C25	155.7 (4)
C7—C8—C9—C12	179.6 (4)	C20—C21—C24—C29	150.5 (4)
C8—C9—C10—C11	−0.7 (6)	C22—C21—C24—C29	−28.1 (5)
C12—C9—C10—C11	−179.4 (4)	C29—C24—C25—C26	−0.8 (6)
C9—C10—C11—C6	−0.2 (6)	C21—C24—C25—C26	175.6 (4)
C7—C6—C11—C10	0.9 (6)	C24—C25—C26—C27	0.9 (7)
C5—C6—C11—C10	−179.4 (4)	C25—C26—C27—C28	0.4 (7)
C8—C9—C12—C17	−148.5 (4)	C26—C27—C28—C29	−1.8 (7)
C10—C9—C12—C17	30.1 (6)	C27—C28—C29—C24	1.9 (7)
C8—C9—C12—C13	32.3 (6)	C25—C24—C29—C28	−0.6 (6)
C10—C9—C12—C13	−149.1 (4)	C21—C24—C29—C28	−177.0 (4)

### Hydrogen-bond geometry (Å, °)

**Table:**

Cg4 is the centroid of the C24–C29 ring.

**Table:**

*D*—H···*A*	*D*—H	H···*A*	*D*···*A*	*D*—H···*A*
C1—H1C···O1^i^	0.98	2.41	3.336 (6)	158
C17—H17A···O3^ii^	0.95	2.47	3.369 (5)	158
C20—H20A···Cg4^iii^	0.95	2.82	3.707 (4)	157

Symmetry codes: (i) *x*, −*y*+1/2, *z*−1/2; (ii) −*x*, −*y*+1, −*z*; (iii) *x*, −*y*−1/2, *z*−3/2.
